# A Novel Pentapeptide Targeting Integrin β3-Subunit Inhibits Platelet Aggregation and Its Application in Rat for Thrombosis Prevention

**DOI:** 10.3389/fphar.2016.00049

**Published:** 2016-03-08

**Authors:** Qingrong Qu, Yamin Liu, Xuejiao Yan, Xiaobo Fan, Naifeng Liu, Guoqiu Wu

**Affiliations:** ^1^Department and Institute of Cardiology, Zhongda Hospital, Medical School of Southeast UniversityNanjing, China; ^2^Center of Clinical Laboratory Medicine of Zhongda Hospital, Institute of Biotechnology and Clinical Pharmacy, Southeast UniversityNanjing, China; ^3^Pharmacy Department of Zhongda Hospital, Southeast UniversityNanjing, China

**Keywords:** peptide, platelet, integrin beta3, blood coagulation, thrombosis

## Abstract

**Background:** Antiplatelet therapy plays a pivotal role in the prevention and treatment of thrombotic diseases. We reported the screening of P1C as a novel integrin-binding peptide from the C-terminal of connective tissue growth factor. Primary study indicated that P1C has potential against platelet aggregation.

**Objectives:** We aimed to find the shortest active unit from the P1C fragments and explore its *in vivo* and *in vitro* activities.

**Methods:** A series of truncated P1C fragments was prepared and screened for antiplatelet activity. The most active fragment was evaluated using coagulation assays. Flow cytometry and confocal microscopy were used to determine the interaction between the peptide and the integrin. The *in vivo* potential was further explored using two types of rat models.

**Results:** From a series of truncated P1C forms, a so-called P1Cm peptide of 5-amino acids, namely, IRTPK was screened out as the shortest active unit with superior activity. Coagulation experiments and an *in vivo* toxicity assay demonstrated that P1Cm is safe *in vivo* and inhibits ADP- and TH-induced human platelet aggregation *in vitro* in a concentration-dependent manner. Furthermore, it has limited effect on the coagulation parameters. Flow cytometry and confocal microscopy experiments consistently indicated that the peptide specifically binds the β3-subunit of integrin on platelets. Further experiments using rat models of artery-vein shunt and carotid arterial thrombosis illustrated that P1Cm can effectively prevent thrombosis formation.

**Conclusion:** P1Cm may be a new, promising antithrombotic alternative to currently available antiplatelet treatments.

## Introduction

Platelet activation, aggregation, and adhesion are considered the general pathogenesis of various cardiovascular disorders, such as acute coronary syndrome, myocardial infarction (De Luca, 2012), stroke, and unstable angina (Rodrigues et al., 2013). These pathological processes are mainly mediated by the receptor of glycoprotein IIb/IIIa or by integrin αIIbβ3 of the platelets (Liu et al., 2005). Each platelet contains 60,000–80,000 molecules of αIIbβ3 protein complexes (Armstrong and Peter, 2012).

Upon platelet activation, αIIbβ3 shifts into a high affinity conformation that efficiently binds the ligands and leads to platelet aggregation, which is considered the classic mechanism of action for all known platelet agonists (Lefkovits et al., 1995). Consequently, blocking the integrin αIIbβ3 receptor is an excellent strategy for the therapeutic intervention for thrombotic diseases (Di Mario, 2014).

Conventional αIIbβ3 inhibitors have helped establish the protocol for antiplatelet therapy in percutaneous coronary intervention (Hanna et al., 2010). Thus far, the United States Food and Drug Administration has approved three αIIbβ3 inhibitors: abciximab, eptifibatide, and tirofiban. However, retrospective analyses obtained through different means have indicated a high risk of bleeding arising from the administration of αIIbβ3 inhibitors (Hechler et al., 2011). Given the importance of antithrombotics in the treatment of ischemic disorders, additional efforts should be directed to searching for novel and better antithrombotic agents.

P1C is a fragment from the C-terminal of connective tissue growth factor, which binds to various integrins such as ανβ3 from endothelial cells (Cornel et al., 2015), αIIbβ3 from blood platelets, and alpha(6)-beta(1) from fibroblast cells (Gao and Brigstock, 2004). The nanoprobe prepared by conjugating P1C with ultra superparamagnetic iron oxide particles confirmed the specific affinity between P1C with ανβ3 in Bel7402 human primary liver cancer cells *in vitro* (Lofblom et al., 2010) and *in vivo* (Wu et al., 2011). However, whether peptide binding to another integrin, such as αIIbβ3, will result in an antagonistic effect on platelet aggregation remains unclear.

In this study, a pentapeptide P1Cm was first screened out from a series of truncated P1Cs with superior activity. The bioactivity of P1Cm was evaluated *in vitro* and *in vivo*.

## Materials and methods

### Peptide synthesis

The P1C peptide and its truncated forms were purchased from Scipeptide (Shanghai, China). The peptides were synthesized by the solid-phase method using a model 432A synthesizer (Applied Biosystems Inc., Foster City, CA; Angiolillo et al., 2004). Molecular mass was determined by electrospray mass spectrometry using LCMS-2010 (Shimadzu, Japan). The purity of the peptide was analyzed using a C18 reverse-phase HPLC (Wu et al., 2008). Table 1 presents a summary of all sequences of the truncated forms.

**Table 1:** **Anti-platelet activity of various peptides and Tirofiban**.

**Peptide/Tirofiban**	**IC50(μM)**
IRTPKISKPIKFELSG (P1C)	80
IRTPK——————(P1Cm)	75
———ISKPI———-	442
—————–KFELSG	408
IRTP——————–	>500
−RTPK——————	>500
−RTP——————–	377
Tirofiban————–	25

### Animals

The animals were obtained from the Laboratory Animal Center, Science Academy of China (Shanghai, China) and raised in an SPF laboratory of a light/dark cycle of 12 h/12 h, with free access to food and water. Animal experiments were carried out according to a protocol approved by the Animal Care and Use Committee of Southeast University, China. To collect fresh animal samples, the animals were sacrificed through an intravenous injection of air after being anesthetized.

### Platelet sample preparation

Blood samples from healthy adult donors who have never received antiplatelet treatment were collected and supplemented with 0.1X volumes of 3.8% trisodium citrate. After centrifugation at 1100 g for 15 min at 22°C, the supernatant was collected as the platelet-poor plasma sample (Oyama et al., 2009). The pellets were transferred into a new tube and further purified by repeated re-suspension and centrifugation in Tyrode's albumin buffer three times. The cell pellets were finally re-suspended in the buffer as the platelet-rich plasma sample. Platelet count was performed using a whole-blood cell counter (Cysmex, USA). All samples were used within 6 h post-collection (Niu et al., 2012).

### Antiplatelet aggregation studies *in vitro*

Platelet aggregation was measured by the turbidimetric assay using a platelet aggregation analyzer (LBY-NJ4, Beijing Precil Instrument Co. Ltd., China; Knight and Romano, 2005). A 300 μL aliquot of platelet-rich plasma with 0–1.20 mM peptide added was incubated under gentle stirring at 37°C for 5 min (Brossi et al., 2015). Adenosine diphosphate (ADP, 20 μmol/L) or bovine thrombin (TH, 200 units/L) was added to induce platelet aggregation. The changes in transparence caused by platelet aggregation were recorded. The transparence of platelet samples with saline added was defined as 100%. The transparence of the platelet sample with ADP/TH added was defined as 0%. The IC50 values of peptides for the blood sample were calculated as mean ± SE.

The activity of P1Cm was further evaluated using the whole blood sample. Briefly, a 500 μL aliquot of whole blood was placed in polystyrene tubes containing 0.009375–0.15 P1Cm. The mixture was stirred at 37°C for 5 min, followed by an addition of 60 μL ADP or an equal volume of saline. Platelet counts were performed using a whole-blood cell counter (Cysmex, USA) after incubation at 37°C for 5 min.

Blood smears were also prepared and stained using the Wright–Giemsa method (Nadal-Wollbold et al., 2010). Smear images were obtained with a Zeiss AxioVert 200M inverted light microscope (Carl Zeiss, Thornwood, NY, USA).

### P1Cm on the coagulation parameters and its acute *in vivo* toxicity

Plasma was collected after centrifugation at 1000 × g for 10 min at room temperature. An aliquot of 100 μl plasma was supplemented with series concentrations of P1Cm, ranging from 0.15 to 0.45 mM, 0.15 mM tirofiban or vehicle (PBS, pH 7.4), followed by an incubation of 5 min at 37°C before being sent to an ACL TOP Automatic Coagulation Analyzer (Beckman Coulter, USA; Wang et al., 2013). The coagulation parameters, such as activated partial thromboplastin time (APTT), prothrombin time (PT), and thrombin time (TT; Chen et al., 2013), were recorded, and each assay was performed in triplicate.

Kunming mice (*n* = 6 for each group) were intravenously injected with different concentrations of P1Cm, ranging from 0.075 to 6 mmol/kg (10x IC90), or tirofiban ranging from 0.025 to 2 mmol/kg (10x IC90). Blood pressure and breath were monitored after injection, and the mesentery were observed under microscope after the animals were sacrificed. Mortality within the next 3 days was recorded.

### Flow cytometry

Washed platelets (1 × 10^6^ platelets/mL) were pre-incubated with anti-human αIIb or β3 monoclonal antibody (5 μg/mL, R&D Systems, Minneapolis, MN) for 10 min at room temperature in the presence of 100 U/L thrombin. After rinsing twice with PBS (Kander et al., 2014), the platelets were co-incubated with 5 μg/mL goat anti-mouse antibody-PE (Caltag Laboratories, USA) and/or 10 μg/mL FITC-conjugated peptide (FITC-P1Cm, synthesized by Science Peptide Bio-Technology Co, LTD., Shanghai, China) at 22°C for 30 min in darkness (Park et al., 2014). The incubations were then washed three times and fixed with 1% paraformaldehyde at 4°C (Kashiwagi et al., 2013) before flow cytometry analysis (Becton Dickinson, San Jose, CA, USA).

### Confocal microscopy

The platelet samples were prepared as previously reported (Mahdi et al., 2002; Elnager et al., 2014; Brzoska et al., 2015). In brief, human platelets (1 × 10^6^ platelets/mL) containing 0.1 mg/mL polylysine (Sigma) were transferred into a 35 mm cell culture dish (Cat no. 627860, Greiner Bio-One, Germany) followed by incubation for 30 min at room temperature. Thereafter, floating platelets were washed away with PBS (pH 7.4). The remaining platelets were treated with 100 U/L thrombin plus 5 μg/mL monoclonal antibody of anti-human αIIb or β3 for 30 min at room temperature. This step was followed by an addition of goat anti-mouse antibody-PE (5 μg/mL) and/or FITC-P1Cm (10 μg/mL) and incubation for 30 min in darkness at 4°C. Finally, the platelets were washed twice with PBS and examined with a confocal laser scanning microscope (OLYMPUS-FV1000, Japan).

### P1Cm against carotid arterial thrombosis

Thirty male Wistar rats (250–300 g) were randomly divided into five groups: high-, middle-, and low-dose treated groups of P1Cm, a saline group, and a tirofiban group (*n* = 6 per group). The rats were operated on the right common carotid artery (Bird et al., 2008). Briefly, the rat was first anesthetized by an intraperitoneal injection of ketamine (100 mg/kg). The fascia was bluntly dissected to expose the right common carotid artery immediately after a dosage of 0.15, 0.30, or 0.45 mmoL/kg of P1Cm, 0.15 mmoL/kg of tirofiban, or an equal volume of saline applied accordingly via tail vein injection. A 3 × 4 mm piece of filter paper saturated with ferric chloride (10% solution) was placed under the right carotid artery for 20 min and then removed. Thrombus size and blood flow were detected by color Doppler flow imaging (CDFI) (13 MHz phased-array transducer, LOGIQ S6 Color Doppler Ultrasonographer, GE, USA; Chua et al., 2012). The animals were kept anesthetized during the CDFI measurements.

### Pathological examination

To obtain fresh samples immediately after measurement, the animals were sacrificed through an intravenous injection of air, and the injured carotid artery was collected for pathological examination. Carotid artery samples were fixed with 10% neutral buffered formalin and embedded in paraffin wax for routine histological analysis with hematoxylin and eosin staining. Morphometric analysis was carried out using a Leica Q500/W microscope (Leica Microsystems, Wetzlar, Germany). The antithrombosis effect was evaluated by calculating the percentage of carotid artery area covered by the thrombus (Hosokawa et al., 2011).

### Preventive efficiency of P1Cm in the artery-vein shunt model

Fifty male Wistar rats (250–300 g) were randomly divided into five groups: high-, middle-, and low-dose groups of P1Cm, a saline group, and a tirofiban group (*n* = 10 per group). The animals were treated with the same concentrations as in the previous thrombosis model, 0.15, 0.30, or 0.45 mmoL/kg of P1Cm, 0.15 mmoL/kg of tirofiban, or an equal volume of saline by tail vein injection after being anesthetized by an intraperitoneal injection of ketamine (100 mg/kg). The operation was carried out as previously reported (Sato et al., 1998; Jing et al., 2011). Briefly, the right carotid and left jugular veins were separated. An 8 cm silk thread was placed inside a polyethylene tube filled with heparin sodium solution (50 IU/mL). The right carotid and left jugular veins were connected with the tube. One end of the tube containing the thread was inserted into the left jugular vein, and the other end was inserted into the right carotid artery. Blood was allowed to flow from the right carotid artery to the left jugular vein through the polyethylene tube for 15 min. The tube was then removed, and the weight of the attached wet thrombus on the thread was measured immediately. The rats were sacrificed through an intravenous injection of air.

### Statistical analyses

Data are expressed as the mean ± SD/SE and analyzed with SPSS 13.0 software. Significant differences between the control and experimental groups were analyzed by one-way analysis (ANOVA) followed by the least-significant difference (LSD) *post-hoc* test. *P* < *0.05* was considered statistically significant.

### Ethics statement

These studies were approved by IEC for Clinical Research of Zhongda Hospital, affiliated to Southeast University (Application number; 2013ZDSYLL109.0).

## Results

### P1Cm activity against platelet aggregation

**Figure 1:**
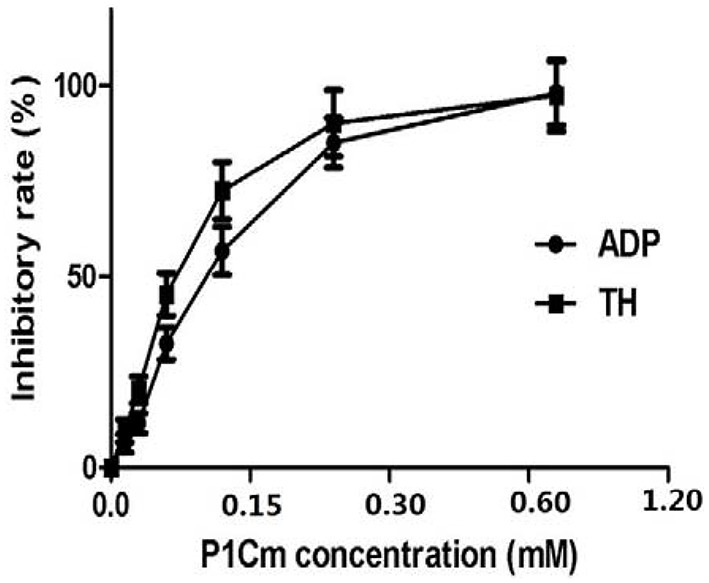
**Effects of P1Cm on ADP- and TH-induced platelet aggregation in human washed platelets**. Platelets were pre-incubated with P1Cm (0–1.2 mM) for 5 min, followed by an addition of ADP (5 μM) or TH (200 units/L). Data are presented as mean ± SE (*n* = 6).

**Figure 2:**
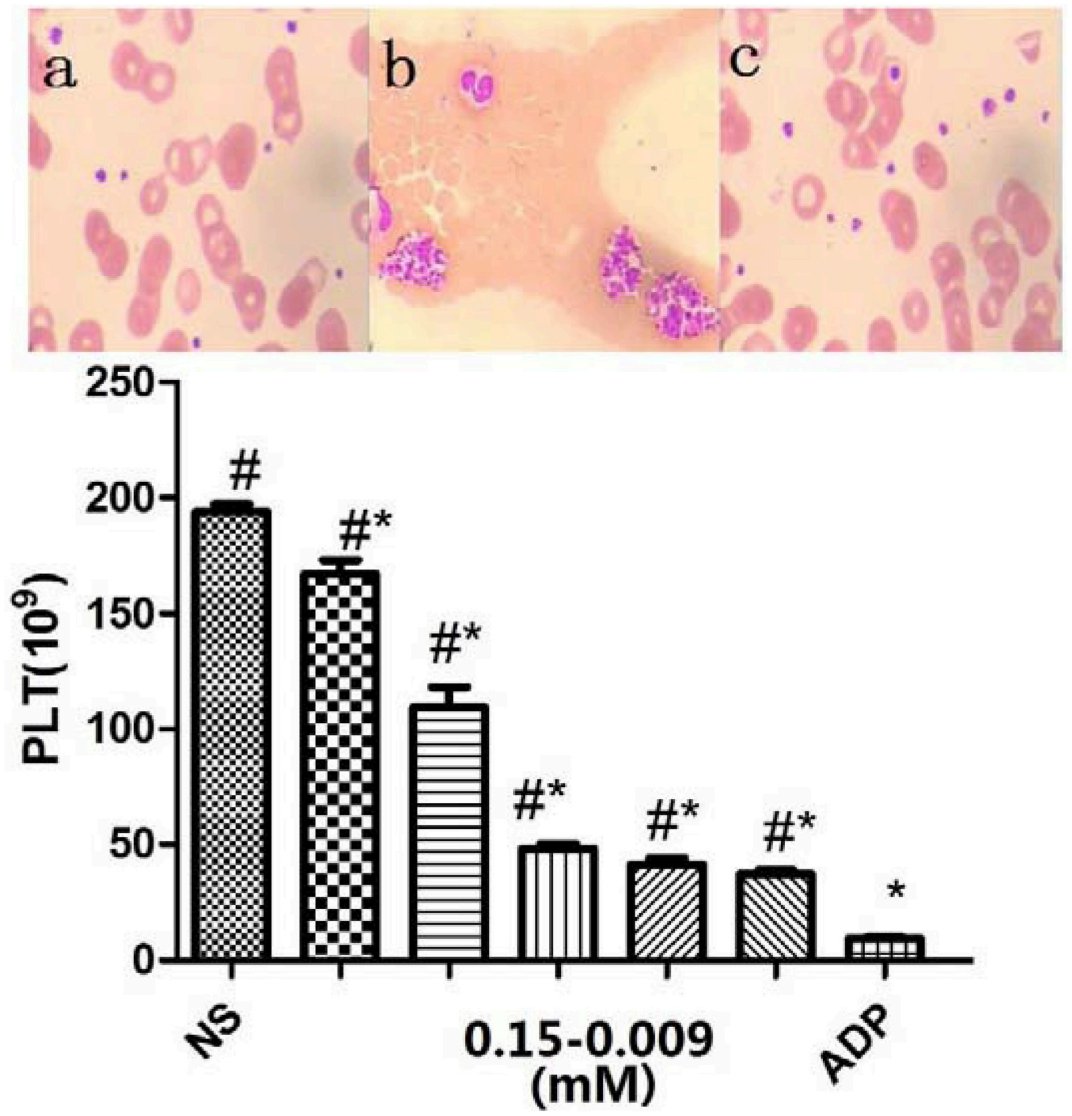
**Inhibitory effects of P1Cm on platelet aggregation in whole blood**. Upper panel, Wright–Giemsa stained blood smears: **(A)** untreated control; **(B)** platelet aggregation by ADP; **(C)** platelets co-incubated with P1Cm (0.075 mM) and ADP. Lower panel, Platelet counts. P1Cm inhibited ADP-induced platelet aggregation at different concentrations by platelet count assay. The blank group was the sample without ADP and P1Cm treatment. Data are expressed as mean ± SM (*n* = 20). ^#^*P* < 0.05 vs. saline group. ^*^P < 0.05 vs. ADP group.

The antiplatelet activities of P1Cm or tirofiban were measured using the turbidimetric assay described above. The IC50 values of P1C and its truncated forms are summarized in Table 1. The smallest unit with antiplatelet activity was a 5-amino acid fragment (IRTPK) termed P1Cm. P1Cm exhibited superior activity to its parent and other truncated P1C forms. P1Cm prevented platelet aggregation in a dose-dependent manner in the platelet-rich plasma samples (Figure 1). ADP induced platelet aggregation in the whole blood sample, as shown in Figure 2B. The whole blood sample pre-treated with 1 mg/ml P1Cm showed a similar diffused distribution of platelets as negative control after the addition of ADP (Figures 2A,C). Respective IC_50_ values for P1Cm inhibiting ADP- and TH-induced platelet aggregation in the washed platelet samples were 145.9 ± 12.13 and 139.8 ± 10.54 μM, and similar results were obtained with the whole blood sample (Figure 2, lower panel).

### Effects on coagulation

**Figure 3:**
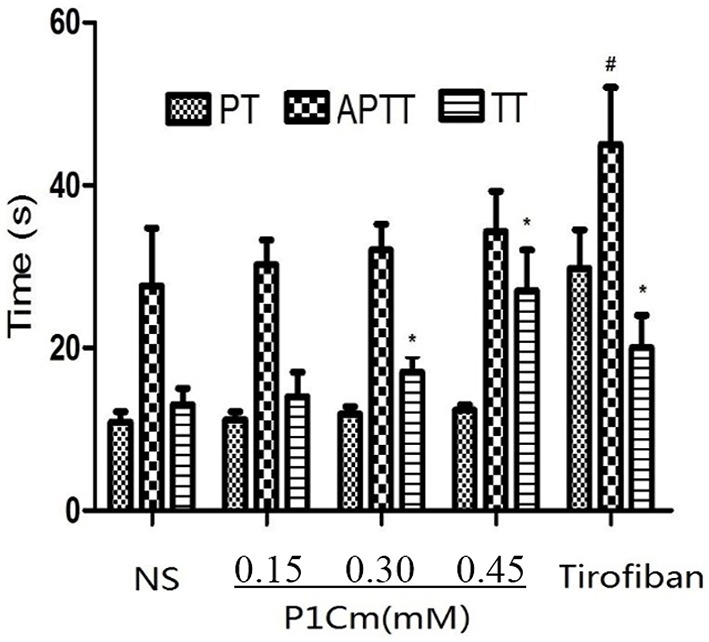
**Effect of P1Cm on coagulation**. ^*^P < 0.05 significantly different from saline group. ^#^Significantly different from the rest of the groups. Data are expressed as mean ± SM (*n* = 6).

The FIB values were 3.61 ± 0.44, 3.82 ± 0.37, 3.94 ± 0.45, 4.07 ± 0.39, and 4.23 ± 0.71 g/L for the saline, P1Cm (low, middle, and high dosage), and tirofiban groups, respectively. The other three parameters are summarized in Figure 3. We observed that TT was postponed, whereas PT and APTT were as normal as the control after P1Cm treatment. P1Cm extended the TT at a dosage-dependent manner. No significant differences between groups of different dosages (high-, middle-, and low-dose groups; *P* > 0.05) were found in PT, APTT, and FIB. In contrast, tirofiban apparently inhibited the coagulation and affected the parameters (*P* < 0.05).

### FITC-P1Cm specifically binds integrin β3-subunits of platelets

**Figure 4:**
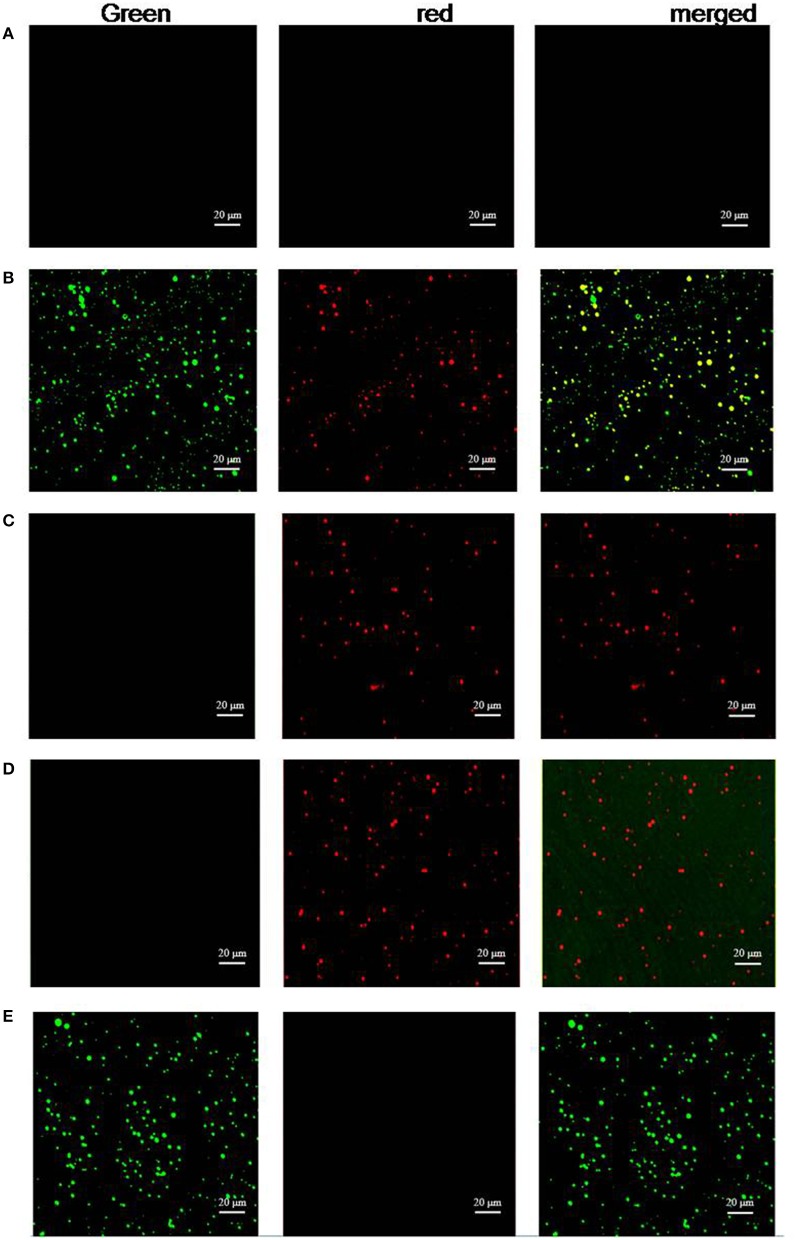
**P1Cm specifically binds to platelets using confocal microscopy**. **(A)** Untreated platelets. **(B)** Platelets pre-treated with anti-αIIb antibody, followed by an addition of goat anti-mouse antibody-PE and FITC-P1Cm. **(C)** Platelets pre-treated with anti-αIIb followed by an addition of goat anti-mouse antibody-PE. **(D)** Platelets were pre-incubated with anti-β3 antibody, followed by the incubation with goat anti-mouse antibody-PE and FITC-P1Cm. **(E)** Platelets were incubated with FITC-P1Cm.

FITC and PE emitted green and red fluorescence, respectively. The platelets incubated with FITC-P1Cm alone showed green fluorescence (Figure 4E). The platelets gave off red fluorescence (Figure 4C) after subsequent treatment with anti-αIIb antibody or anti-β3 antibody and goat anti-mouse antibody-PE. Only red fluorescence was present when the platelets were pre-incubated with β3 antibody before FITC-P1Cm treatment (Figure 4D). In contrast, both green and red fluorescence were observed when the platelets were subsequently treated with anti-αIIb antibody, goat anti-mouse antibody-PE, and FITC-P1Cm (Figure 4B). No fluorescence signal was found in blank platelets without any treatment (Figure 4A).

**Figure 5:**
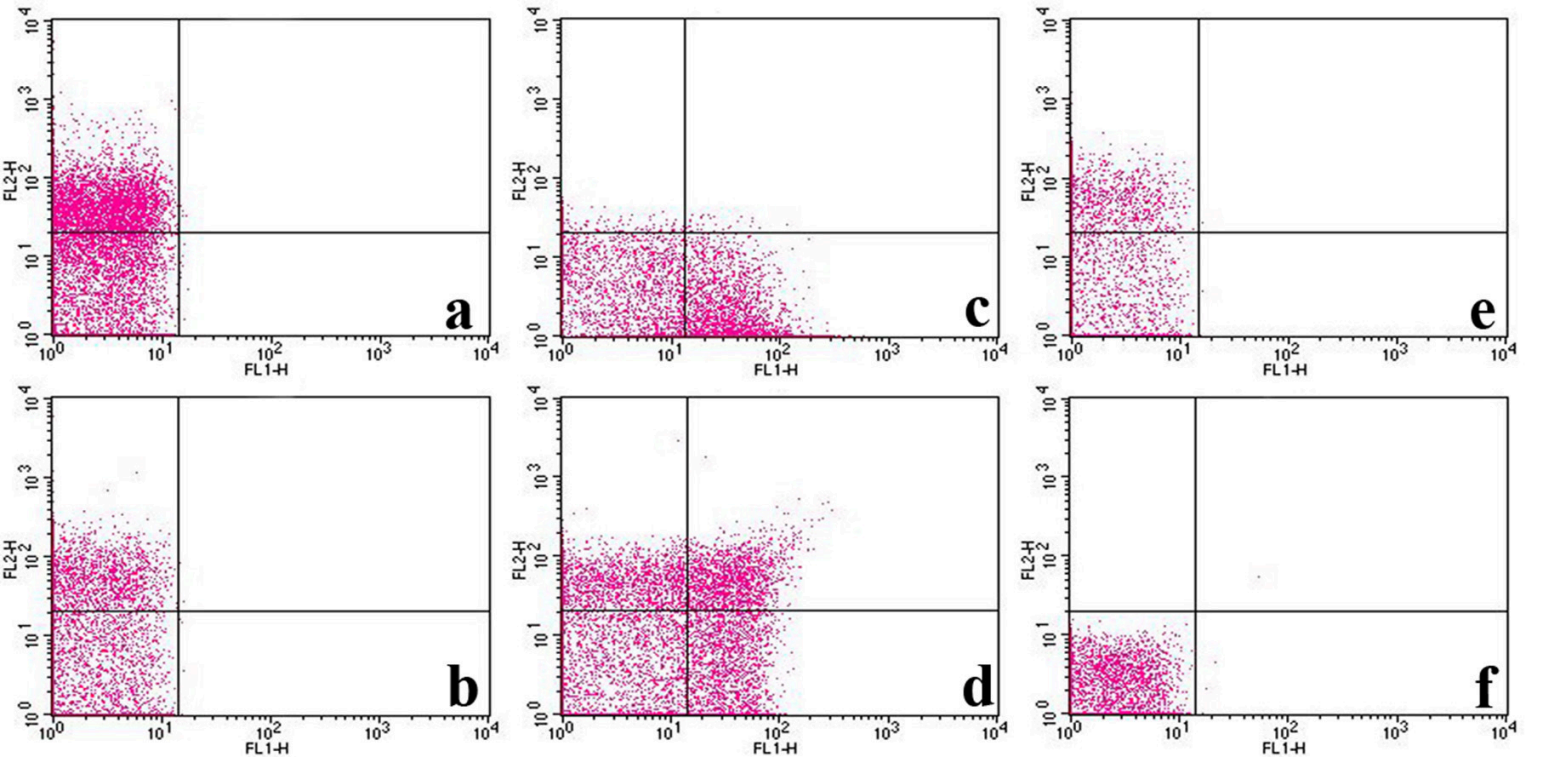
**FITC-labeled P1Cm specifically binds to platelets using flow cytometry**. **(A)** Platelet sample treated with anti-αIIb antibody and goat anti-mouse antibody-PE successively. **(B)** Sample treated with anti-β3 antibody and goat anti-mouse antibody-PE. **(C)** Sample treated with FITC-P1Cm. **(D)** Sample pre-treated with anti-αIIb antibody, followed by an addition of goat anti-mouse antibody-PE and FITC-P1Cm. **(E)** Samples pre-incubated with anti-β3antibody, followed by an addition of goat anti-mouse antibody-PE and FITC-P1Cm. **(F)** Untreated platelets.

Flow cytometric assay was performed to further confirm the affinity between P1Cm with the β3-subunit. Flow cytometry was used to determine the expression of αIIb/β3-subunits of integrin and their interaction with FITC-labeled P1Cm. As shown in Figure 5, the αIIb- and β3-expression in platelets were 70.23 ± 2.34 and 50.18 ± 4.86%, respectively, with fluorescence signals located in the upper left quadrant (PE red; Figures 5A,B). The signal of the P1Cm-FITC bound platelets was 47.50 ± 5.21%, as shown in the lower right quadrant (FITC green; Figure 5C). No competition was observed between anti-αIIb mAb with P1Cm for platelet binding because the FITC green channel signal barely changed after receiving extra anti-αIIb mAb (50.45 ± 7.31 vs. 47.50 ± 5.21%). Some of the platelets were also double-stained (Figure 5D) after being co-incubated with P1Cm-FITC and anti-αIIb mAb. Consistent with the confocal microscopy result, the platelets pre-incubated with β3 antibody no longer showed affinity with P1Cm, as shown in Figure 5E, indicating that anti- β3 competed with P1Cm for the same target. An untreated platelet sample was used as the blank control (Figure 5F).

### *In vivo* antithrombotic activity

**Figure 6:**
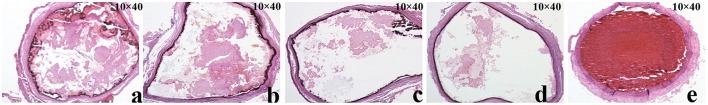
**Pathological images of thrombosis in carotid artery**. Slices of carotid artery after rats received **(A)** 0.15 mmol/kg P1Cm, **(B)** 0.30 mmol/kg P1Cm, or **(C)** 0.45 mmol/kgP1Cm, **(D)** 0.15 mmol/kg tirofiban, or **(E)** saline.

CDFI was used to detect the thrombosis size and evaluate the stenosis of the blood vessel through peak systolic flow velocity (PSFV). Anti-coagulation treatments significantly relieved the thrombosis. PSFV was negatively related to the vascular stenosis rate (VSR). When the vessel was blocked by thrombus, the flow rate increased as compensation for the supply of sufficient blood to the tissue. As expected, the highest P1Cm dosage resulted in the slowest PSFV and the smallest VSR (Figure 6, Table 2), and its effects were very close to those of tirofiban on PSFV and VSR. The thrombosis areas in the carotid artery were 0.173 ± 0.004, 0.077 ± 0.004, and 0.0162 ± 0.002 cm^2^ for the saline, low-dose, and middle-dose groups, respectively, but these were not detected in the high-dose group (Table 2, Figure S1 and Figure 6). P1Cm prevented thrombosis in a concentration-dependent manner, and a difference was observed within the P1Cm groups in thrombosis size, VSR, and PSFV (*P* < 0.01). The vessel of the saline-treated model group was almost blocked without PSFV detection.

**Table 2:** **Effects of P1Cm on the carotid thrombosis model and the artery-vein shunt model**.

**Groups (mmol/kg)**	**PSFV (cm/s)**	**SIZE/area (cm^2^)**	**EMW (mg)**	**VSR (%)**
Saline	None^#^	0.173 ± 0. 004	46.07 ± 4.46	83.95 ± 5.91
Tirofiban 0.15	84.2 ± 3.50^*^	0.007 ± 0.001^*^	27.51 ± 4.03^*^	28.25 ± 5.83^*^
P1Cm 0.15	109.6 ± 10.52^*^	0.077 ± 0.004^*^	41.77 ± 5.87	66.05 ± 6.99
P1Cm 0.30	97.3 ± 3.22^*^	0.016 ± 0.002^*^	36.83 ± 4.86^*^	50.00 ± 8.50^*^
P1Cm 0.45	81.5 ± 5.59^*^	None^+^	26.10 ± 4.74^*^	31.84 ± 12.72^*^

*Thrombosis size (Size/area), peak systolic flow velocity (PSFV), and vascular stenosis rate (VSR) were measured after administering P1Cm at a dose of 0.15–0.45 mmol/Kg in the ferric chloride-induced carotid thrombosis models (n = 6). Thrombosis wet weights (EMW) were measured after administering P1Cm at a dose of 0.15–0.45 mmol/Kg in the artery-vein shunt models (n = 10). Data are expressed as mean±SD*.

# *Blood vessel was almost blocked by thrombus*.

+ *No thrombus was detected*.

* *P < 0.05 vs. saline groups*.

In the artery-vein shunt model, thrombus formation was prevented by antiplatelet treatment (Table 2). The thrombus weights evidently decreased by 9.33, 20.04, 43.35, and 39.92% from the P1Cm (low 0.15 mM, middle 0.30 mM, and high dosage 0.45 mM) and tirofiban 0.15 mM groups, respectively, in comparison with those from the saline group. The difference was statistically significant between the saline and treated groups (*P* < 0.05), except for the P1Cm low-dose group (*P* > 0.05).

As shown in Table 3, both P1Cm and tirofiban seemed safe when IC90 or a lower dosage was applied. P1Cm seemed to be safer than tirofiban when a higher dosage of 10X IC90 was applied. In contrast to the effects of P1Cm, the mice showed motor retardation after receiving 10X IC90 of tirofiban, and two mice died within 16 h. Pathological examination showed the mesentery was characterized by local hemorrhage.

**Table 3:** **Acute toxicity of P1Cm and Tirofiban in mice (*n* = 6)**.

**Dosage/mortality**	**IC50**	**IC90**	**10x IC90**
P1Cm	0	0	0
Tirofiban	0	0/6	2/6

## Discussion

Aberrant platelet activation may cause thrombosis and eventually lead to serious vascular symptoms, such as cerebral stroke and myocardial infarction (De Luca, 2012). As an essential molecule during platelet activation and aggregation, αIIbβ3 is an excellent target for therapeutic intervention in thrombotic diseases (Nicholls et al., 2012; Diamond, 2013).

We reported on P1C as an integrin-targeting peptide. In this study, a pentapeptide (IRTPK), P1Cm with antiplatelet aggregation, was screened out from a series of P1C truncated forms (Table 1). Numerous substances can target integrin, but few studies report on the discrimination of the two subunits of integrin for targeting. Through flow cytometry and confocal microscopy analyses, we demonstrated that P1Cm was targeted on the β3-subunit. Reports have indicated that the soluble form of the CD40 ligand, a tumor necrosis factor family member, mainly expressed on activated T-cells and platelets can mediate platelet stimulation by binding αIIbβ3 through a KGD domain (Prasad et al., 2003; Lonsdorf et al., 2012). The β3-subunit is enriched on the platelet surface, an outcome that is the theoretical basis of the present study.

In the model of carotid thrombosis induced by ferric chloride, P1Cm inhibited arterial thrombus formation in a concentration-dependent manner. The CDFI results illustrated that the thrombosis size and PSFV from the mid- and high-dose groups were statistically different compared with those from the saline group (*P* < 0.05). Significant differences were also observed between the three P1Cm dosage groups (*P* < 0.05). The result of pathological examination was in accordance with that of CDFI. The artery-vein shunt thrombosis model also showed the efficiency of P1Cm to reduce thrombus weight.

Our primary results indicated the weak effect of P1Cm *in vivo* when used at the concentration of IC50; thus, 2x IC50 was chosen as the lowest concentration for the *in vivo* experiments. In this study, P1Cm showed 43.35% thrombotic inhibition at a dose of 0.45 mmol/kg, which was equal or even superior to the effect of tirofiban at the dose of 0.15 mmol/kg, as shown by the *in vivo* experiment. Importantly, P1Cm seemed to be safer than tirofiban, as shown by the coagulation assay and *in vivo* toxicity evaluation. P1Cm had less effect on the extrinsic and intrinsic coagulation cascades. PT and APTT are usually used to evaluate the extrinsic and intrinsic coagulation pathways, respectively. In contrast to tirofiban, no significant difference in PT or APTT was found between the P1Cm-treated groups (high, middle, and low doses), and the saline group (*P* > 0.05) in this study. The lethal dose of P1Cm overreached 10X IC90 of P1Cm, whereas tirofiban caused mouse death at the dosage of 10X IC90. Histological examination indicated that tirofiban caused capillary vessel hemorrhage.

In the last decades, millions of thrombosis patients have benefited from antiplatelet treatments, including αIIbβ3 inhibitors, and most have survived. Nevertheless, these patients were confronted with another risk of intrinsic bleeding, which raised the paradox of the antiplatelet substance being able to inhibit the thrombosis efficiently but not affecting the normal coagulation. Given that platelets play an essential role in hemostasis and thrombosis (Abtahian et al., 2015), balancing platelet function is effective in preventing thrombosis and seems to be the key to reducing bleeding risk during antiplatelet therapy (Viswanathan et al., 2012). In fact, because of the high risk of bleeding due to their unconscionable high affinity to αIIbβ3, receptor inhibitors are no longer considered clinically safe (Elcioglu et al., 2012). The occurrence of thrombocytopenia and the increased risk of major and minor bleeding complications have become main concerns. Thrombocytopenia is frequently reported for abciximab, eptifibatide, and tirofiban treatment, and bleeding complications are regarded as major determinants of clinical outcomes in percutaneous coronary interventions (Huynh et al., 2005). However, αIIbβ3 inhibitors are still widely used to prevent periprocedural thrombosis during PCI. Thus, the need for novel, safe antithrombotic drugs is urgent.

Balancing the risk of thrombocytopenia or bleeding complications with the curative effect of anti-coagulants is a difficult undertaking (Ji and Hou, 2011). The key lies in the affinity of P1Cm to αIIbβ3. One possible solution is to administer P1Cm by continuous infusion. P1Cm may alleviate the risk of thrombocytopenia and bleeding complications, as it shows relatively smaller efficiency than other αIIbβ3 inhibitors. Cyclization is another option to improve the affinity. We plan to focus on modifying the structure to further develop P1Cm.

In addition, unlike eptifibatide from snake venom, P1Cm is a small peptide derived from human connective tissue growth factor, and it is expected to cause very limited allergic or hypersensitivity reactions during or after infusion. Compared with antibodies such as abciximab, P1Cm is a small peptide of 5-amino acid that results in less stereo-specific blockades, and possibly a high drug-to-receptor ratio. In reference to the results on toxicity *in vivo* (Table 3), the safety of P1Cm is apparently superior to, but not as effective as, that of tirofiban.

In conclusion, P1Cm can clearly inhibit ADP- or TH-induced human platelet aggregation *in vitro* in a concentration-dependent manner by specifically binding to the β_3_-subunit. Compared to tirofiban, P1Cm seems to be safe when a much higher dosage is applied *in vivo*, with less effect on the extrinsic and intrinsic coagulation pathways. Further experiments using two rat thrombosis models demonstrated that P1Cm is also an excellent antithrombotic agent *in vivo*. P1Cm is a promising antithrombotic alternative to currently available treatments.

## Author contributions

QQ and GW performed most of the experiments and prepared the manuscript. YL assisted with the rat experiments. XF revised the manuscript. GW and NL were the group leaders from two independent groups offering supervision and financial support.

### Conflict of interest statement

The authors declare that the research was conducted in the absence of any commercial or financial relationships that could be construed as a potential conflict of interest.

## Supplementary material

Figure S1 **PSFV and thrombosis size in ferric chloride-induced carotid thrombosis models detected by CDFI**. Rats were measured after receiving **(A)** 0.15 mmol/Kg, **(B)** 0.30 mmol /Kg or **(C)** 0.45 mmol/Kg P1Cm, or **(D)** 0.15 mmol/Kg tirofiban. Saline **(E)** was used as negative control.
