# Supplementary figures and images for: A Novel Pentapeptide Targeting Integrin β3-Subunit Inhibits Platelet Aggregation and Its Application in Rat for Thrombosis Prevention
Source: Front Pharmacol. 2016 Mar 8;7:49. doi: 10.3389/fphar.2016.00049 (PMC4782163; doi:10.3389/fphar.2016.00049)

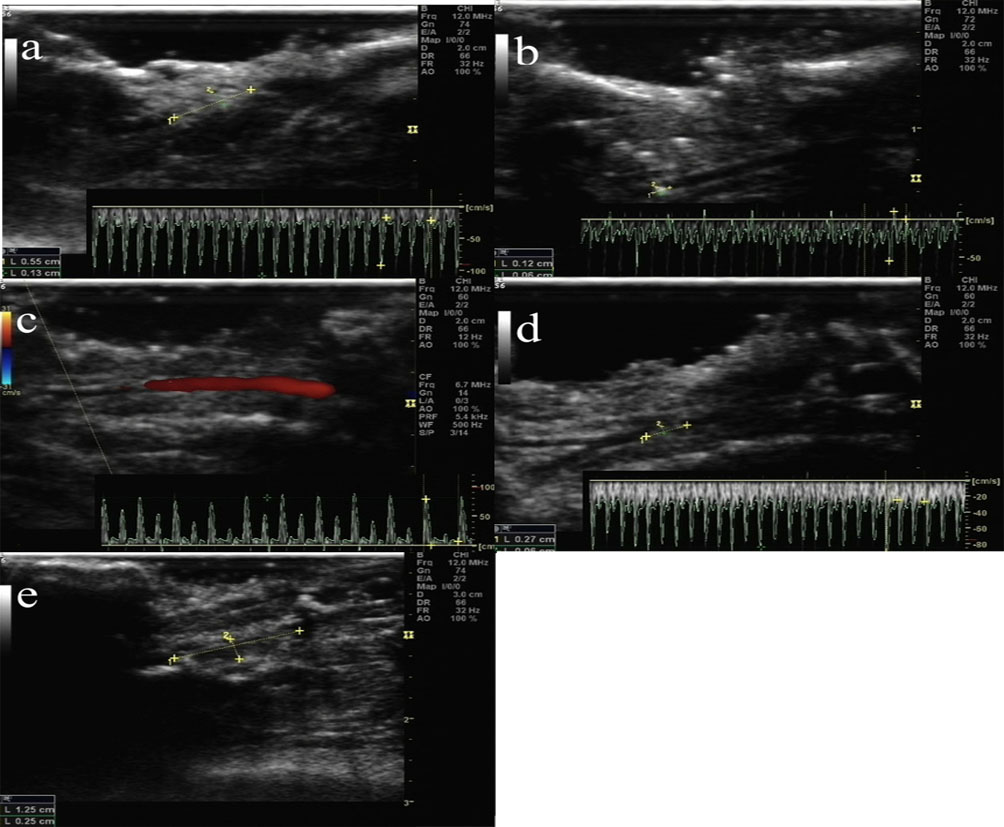

Supplement: Figure S1 — PSFV and thrombosis size in ferric chloride-induced carotid thrombosis models detected by CDFI. Rats were measured after receiving (A) 0.15 mmol/Kg, (B) 0.30 mmol /Kg or (C) 0.45 mmol/Kg P1Cm, or (D) 0.15 mmol/Kg tirofiban. Saline (E) was used as negative control. [file Image1.JPEG]
